# Endocan Is an Independent Predictor of Heart Failure-Related Mortality and Hospitalizations in Patients with Chronic Stable Heart Failure

**DOI:** 10.1155/2019/9134096

**Published:** 2019-04-04

**Authors:** Gorazd Kosir, Borut Jug, Marko Novakovic, Mojca Bozic Mijovski, Jus Ksela

**Affiliations:** ^1^Department of Cardiac Surgery, University Medical Centre Maribor, Maribor, Slovenia; ^2^Department of Vascular Diseases, Division of Internal Medicine, University Medical Centre Ljubljana, Ljubljana, Slovenia; ^3^Faculty of Medicine, University of Ljubljana, Ljubljana, Slovenia; ^4^Department of Cardiovascular Surgery, University Medical Centre Ljubljana, Ljubljana, Slovenia

## Abstract

**Background:**

Heart failure (HF) is characterized by unfavorable prognosis. Disease trajectory of HF, however, may vary, and risk assessment of patients remains elusive. In our study, we sought to determine the prognostic impact of endocan—a novel biomarker of endothelial dysfunction and low-grade inflammation—in patients with heart failure.

**Methods:**

In outpatients with chronic HF, baseline values of endocan were determined and clinical follow-up for a minimum of 18 months obtained. A multivariate Cox proportional hazard model was built for HF-related death or hospitalization requiring inotropic support.

**Results:**

A total of 120 patients (mean age 71 years, 64% male, mean LVEF 36%) were included. During a mean follow-up of 656 ± 109 days, 50 patients (41.6%) experienced an event. On Cox multivariate analysis, endocan values emerged as an independent predictor of HF prognosis (HR, 1.471 CI 95% 1.183-1.829, *p* = 0.001, for each 1 ng/mL increase) even after adjustment for age, gender, HF etiology, LVEF, NYHA class, NT-proBNP, and exercise tolerance.

**Conclusions:**

Endocan is an independent predictor of HF-related events in chronic HF individuals and represents a promising tool for risk assessment of HF patients.

## 1. Introduction

Heart failure is characterized by unfavorable prognosis [1–3]. Patient populations, however, may differ strikingly in terms of etiology, pathophysiology, and natural disease progression [1, 3]. With the rising prevalence of heart failure worldwide, accurate risk stratification is becoming increasingly important, as it may help identify patients in need of intensified medical treatment, stringent follow-up, and/or advanced heart failure therapies (such as left ventricular assist device and/or heart transplant) and devote healthcare resources to those HF individuals, which need it the most. Identification of prognostic biomarkers to improve risk stratification of heart failure patients beyond traditional clinical predictors, such as etiology, left ventricular ejection fraction (LVEF), or New York Heart Association (NYHA), has therefore become pivotal in tackling the ongoing heart failure epidemics [1, 4, 5].

Endocan is a novel biomarker, which has been closely associated with endothelial dysfunction and low-grade inflammation [6–8]. As unfavorable prognosis in heart failure is at least partially driven by endothelial dysfunction and low-grade inflammation, endocan represents a promising tool for detection of peripheral derangement and their prognostic impact in individuals with heart failure [4, 9–12]. A growing body of evidence suggests that endocan plays an important role in vascular contribution to organ-specific inflammation and endothelial-dependent pathological processes through the regulation of cell adhesion and proliferation in various vascular diseases, inflammation disorders, and angio-, neo-, and tumorigenesis [6–8, 13–16]. Endocan has established itself as a promising inflammatory marker of endothelial dysfunction and a promising indicator of morbidity and mortality in various pathologies, such as sepsis [17, 18]; assorted cardiovascular, lung, and kidney diseases [7, 13, 14, 19, 20]; different malignancies [8, 15, 16]; and preeclampsia [21]. The prognostic impact of endocan in heart failure, however, has not been appreciated to date [22].

The aim of the present study was to describe baseline values of endocan in individuals with chronic HF and to evaluate the potential prognostic impact of the novel biomarker on morbidity and mortality in ambulatory HF patients.

## 2. Materials and Methods

### 2.1. Patient Population

**Figure 1:**
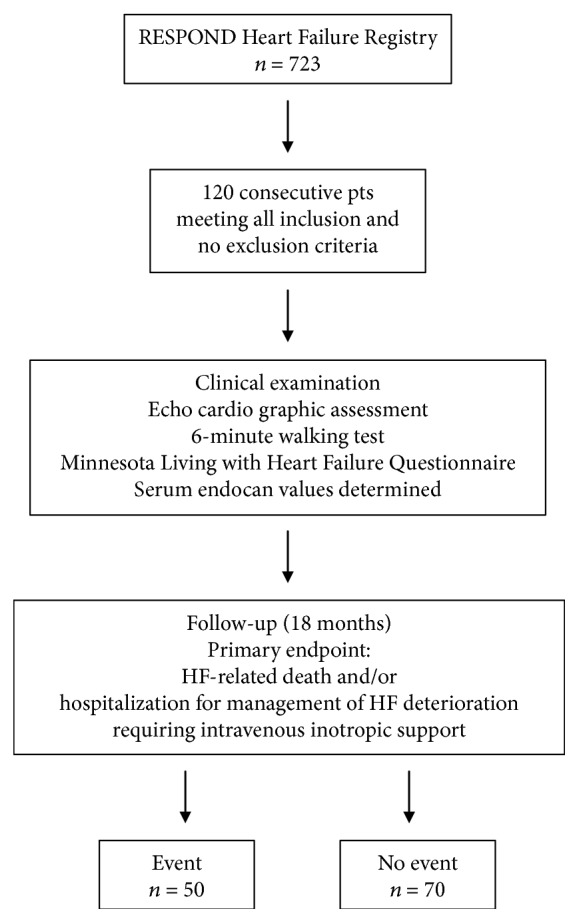
Patient flow diagram.

One hundred and twenty consecutive chronic HF patients from the RESPOND Heart Failure Registry at the University Medical Centre Ljubljana were recruited for the study (Figure 1), as previously reported [23]. The National Ethics Committee approved the study protocol, and the study was conducted according to the Declaration of Helsinki. All patients gave a written informed consent prior to the enrollment.

Patients were included if they met the following criteria: (1) signs/symptoms of HF at inclusion, (2) echocardiographic evidence of left ventricular dysfunction with reduced LVEF (≤49%) or preserved LVEF (>50%) with either an *E*/*E*′ ratio on tissue Doppler recordings at the mitral ring > 15 or an *E*/*E*′ratio > 8 if concomitant AF is present or if natriuretic peptides are elevated or if echocardiographic indices of diastolic dysfunction on transmitral and pulmonary vein flow pattern exist, (3) NYHA class II or III, (4) optimal medical therapy according to the current ACC/AHA and European guidelines, and (5) stable disease for at least 3 months prior to inclusion. Patients were excluded if they met one of the following criteria: (1) less than 3 months from the last myocardial infarction, stroke, or thromboembolic event; (2) severe liver dysfunction (>3 times the upper reference limit of liver enzymes); (3) severe renal dysfunction (creatinine level > 250 *μ*mol/l); (4) acute or chronic autoimmune or inflammatory disease, or (5) known malignancies.

### 2.2. Study Design

At inclusion, patients underwent thorough clinical examination, comprehensive echocardiographic assessment, and 6-minute walking test (6MWT) and completed the Minnesota Living with Heart Failure (MLHF) Questionnaire. Coronary artery disease (CAD) was defined as an angiographically proven obstructive atherosclerotic lesion ≥ 50% of at least one subepicardial coronary artery; peripheral arterial disease (PAD) was defined as obstructive disease of the peripheral arteries proven by computed tomography or angiography or brachial ankle index < 0.9; arterial hypertension (AH) was defined as systolic pressure ≥ 140 mmHg or diastolic pressure ≥ 90 mmHg or intake of antihypertension therapy; diabetes mellitus (DM) was defined as glycemia ≥ 7 mmol/L after a 6-hour fasting period or ≥11 mmol/L two hours postprandial or intake of antiglycemic agents; and hyperlipidemia (HLP) was defined as total cholesterol levels ≥ 4.0 mmol/L or LDL ≥ 2.0 mmol/L or intake of antilipemic drugs and kidney insufficiency (KI) as calculated glomerular filtration rate (GFR) using CKD-EPI equation < 60 mL/min/1.73 m^2^. Venous blood samples were taken in the morning hours after overnight fast from the cubital vein. Blood samples were centrifuged at 3000 rpm for 10 minutes at 0°C, separated immediately afterwards, and stored at -80°C for further use.

### 2.3. Endocan Measurement

Endocan plasma levels were determined in an independent laboratory blinded to the patient clinical data on stored plasma specimens using a commercially available, sandwich-based enzyme-linked immunosorbent assay (ELISA; Lunginnov® Systems, Lille, France) following the manufacturers' instructions. This assay quantitatively measures the concentration of endocan in EDTA plasma and has been shown to have high sensitivity with a lower detection limit of 0.2 ng/mL, linearity between 0.4 and 10.0 ng/mL, no cross-reactivity with other human proteoglycans, and no known interference by commonly used heart failure medications.

### 2.4. Follow-Up Protocol

All patients were followed on the outpatient basis at the Heart Failure Clinic and evaluated by a dedicated cardiologist for a minimum of 18 months at regular 3-month interval visits. If the patient missed a follow-up appointment, a telephone contact with him/herself or his/her relatives or the general practitioner was carried out and all relevant medical records examined in order to assess any changes in patient's health status. The primary outcome in our study was the composite of HF-related death (pump failure or sudden cardiac death) and/or unplanned hospitalization for management of HF deterioration requiring intravenous inotropic support. In all events, the observed composite endpoint was reconfirmed by two additional independent cardiologists, blinded for baseline measurements.

### 2.5. Statistical Analysis

Baseline characteristics are expressed as mean ± standard deviation for normally distributed, as median (interquartile range) for nonnormally distributed continuous variables, and as frequency (percentage) for categorical variables. Between-group differences were appraised by a *t*-test for normally distributed variables and by the Mann-Whitney *U* test for nonnormally distributed variables, and proportions were compared using the *χ*^2^ test. Kaplan-Meier curves and a log-rank test were used to evaluate event-free survival. Cox proportional hazard models were constructed to assess prognostic significance of established HF parameters and plasma endocan levels and were expressed as hazard ratio (HR) with corresponding 95% confidence intervals (CI). Two-tailed *p* values equal or less than 0.05 were considered statistically significant. Obtained data set was statistically evaluated using SPSS Statistics version 23 (SPSS Inc., Chicago, USA).

## 3. Results

### 3.1. Study Population

A total of 120 patients were enrolled and data from all included individuals was utilized in the final analysis. In our study cohort, the mean age was 71 ± 11 years; 64% of all patients were male, and mean LVEF was 35.5 ± 12.8%. Mean follow-up time was 656 ± 109 days. In that period, 50 patients (41.6%) experienced an event (HF-related death or hospitalization requiring intravenous inotropic support). During follow-up, only minor changes in patient's therapy were performed, i.e., uptitration of RAAS inhibitors in 2 individuals and modification of diuretics in 13 patients. Patients who experienced an event had significantly lower LVEF, lower exercise tolerance assessed by 6MWT, higher NT-proBNP, and higher NYHA class; additionally, they significantly likely had ischemic etiology of HF. Baseline clinical and laboratorial characteristics of our patient cohort are depicted in Table 1.

### 3.2. Plasma Endocan Values in Chronic Ambulatory HF Patients

**Figure 2:**
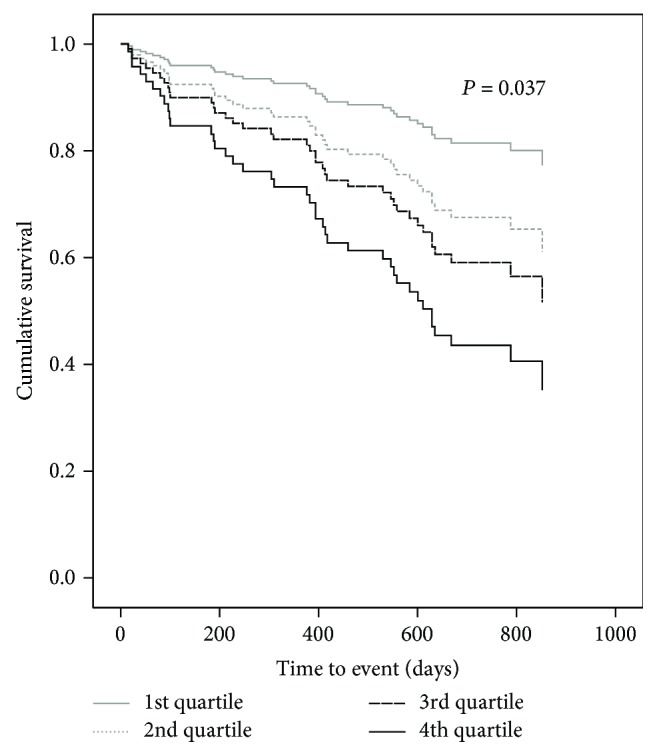
Cumulative HF event-free survival curves according to baseline endocan levels expressed in quartile cut-offs.

Median plasma endocan levels in our patient cohort was 3.38 (2.46-4.81) ng/mL (Table 1) with none of the recorded patient's comorbidities significantly influencing endocan levels (Table 2). However, individuals experiencing an HF-related event had significantly higher values of plasma endocan as compared to HF patients not experiencing an event during the follow-up period (4.26 (3.16-6.13) ng/mL vs. 3.21 (2.25-4.45) ng/mL, *p* < 0.001) (Table 1). In Kaplan-Meier survival analysis, our patients experienced the primary endpoint earlier and more frequently if determined plasma endocan levels were in a higher quartile (mean for survival time ± SD: 797 ± 52 vs. 750 ± 44 vs. 730 ± 62 vs. 559 ± 63 days, *p* = 0.037, for the 1^st^ vs. 2^nd^ vs. 3^th^ vs. 4^th^ quartile, respectively) (Figure 2).

### 3.3. Prognostic Impact of Endocan in Chronic Ambulatory HF Patients

An adjusted Cox proportional hazard model was built to test endocan values as a predictor of HF related death and/or unplanned hospitalization for management of HF deterioration requiring intravenous inotropic support (HR, 1.518 CI 95% 1.269-1.816, *p* < 0.001). Endocan retained its prognostic value also after adjusting for established HF parameters (namely, age, gender, ischemic HF etiology, NYHA classification, LVEF, NT-proBNP, and 6MWT) in a multivariate analysis (HR, 1.471 CI 95% 1.183-1.829, *p* = 0.001) (Table 3).

## 4. Discussion

The main findings of our study are that (i) plasma levels of endocan—a novel marker of endothelial dysfunction—are markedly elevated in patients with chronic HF as compared to previously reported values in healthy subjects or patients with CAD; (ii) endocan levels are increased irrespective of comorbidities, such as CAD, PAD, AH, DM, KI, or HLP; and (iii) plasma endocan levels are an independent predictor of HF-related morbidity and mortality. Our findings suggest that the extent of endothelial dysfunction—as determined by endocan levels—could potentially assist in prognostic assessment and risk stratification of HF patients and thus may potentially help identify patients who would mostly benefit from intensified medical treatment, stringent follow-up, and/or advanced heart failure management options.

Chronic HF is becoming one of the most prominent public health problems affecting around 2% of general and over 10% of elderly populations and rising in prevalence each year [1–3]. Worldwide, mortality among affected individuals exceeds 20% within 1 year after first hospital or outpatient admission and the majority of patients die within 5 years from the diagnosis [1, 2]. As heart failure represents an end-stage syndrome of virtually any cardiac condition, it is characterized by varying etiologies, pathophysiologies, clinical trajectories, and prognostic ramifications [1–3]. Although numerous studies have identified selected clinical parameters such as age, gender, HF etiology, LVEF, NYHA class, and NT-proBNP level as independent predictors of adverse HF prognosis [1, 2], considerable data suggests that objective measure of subclinical disease and pathophysiological derangements related to HF, such as neurohumoral activation, endothelial dysfunction, or low-grade inflammation, may provide estimation of morbidity and mortality risk beyond clinical parameters [4, 9, 12, 23–25]. The blunt distinction between heart failure with preserved and reduced ejection fraction, for example, has recently been challenged in terms of similarly ominous prognosis for the two conditions [22], which clearly reflects the inadequacy of simplified prognostic criteria, like LVEF, to adequately address the complex peripheral and systemic derangements in heart failure population. Plasma biomarkers derived from HF-related pathophysiological derangements—such as endocan—therefore represent a particularly promising tool for the prognostic assessment of heart failure [4, 9, 23–25].

Over the last two decades, a vast amount of evidence has accumulated that endothelial dysfunction plays a pivotal role in the development and deterioration of HF with various endothelial functions, including vasomotor, hemostatic, antioxidant, and inflammatory activity being affected [10–12, 26]. Although substantial differences exist in the pattern of endothelial dysfunction depending on etiology, severity, and stability of HF, endothelial abnormalities seem to be a common feature of this complex syndrome [10–12]. Endothelial health has commonly been assessed by established vascular functional tests, all of which have a tendency to be either invasive or notably operator-dependent [10, 12]. In recent years, noninvasive measures of selected biomarkers such as E-selectin or von Willebrand factor have been introduced in order to objectively describe endothelial derangements [10–12]. However, thus far, none of the used biomarkers has shown proper correlation to conventional clinical or laboratory parameters of HF severity (i.e., LVEF, 6MWT, NYHA class, and NT-proBNP levels) or the ability to consistently differentiate between stable HF patients and those who will more likely deteriorate in short-, mid-, or long-term [10, 12]. To the best of our knowledge, the present study is the first to describe plasma values of a novel biomarker of endothelial dysfunction endocan in chronic HF patients. Unsurprisingly, endocan values were elevated in our patient cohort as compared to previously reported values in healthy individuals and patients with CAD, which was anticipated given the fact that HF is known to disturb normal endothelial activity in a greater extent than isolated CAD [19]. Interestingly, endocan was overexpressed independently from eventual patients' comorbidities such as CAD, PAD, AH, KI, or HLP—all pathologies that are known to have an unfavorable impact on endothelial health per se [7, 8, 13–21, 27]. Given that there is an ongoing debate whether endothelial disturbances in HF are an epiphenomenon related to concomitant pathologies and comorbidities (such as atherosclerosis, metabolic disturbances, or renal insufficiency) or an intrinsic determinant of HF [1, 4, 9, 10, 23–25], our findings clearly indicate that endothelial dysfunction in an HF setting is predominantly driven by pathophysiological derangements of HF itself and cannot be primarily attributed to the concomitant atherosclerotic, metabolic, and/or renal processes of concurrent diseases. Additionally, endocan has shown the ability to differentiate between patients remaining in a stable form of HF and those with deterioration of their HF status leading to death or unfavorable hospitalization requiring inotropic support. Furthermore, endocan also correlated well with clinically applicable indicators of HF progression, as our patients reaching the composite endpoint had also significantly lower LVEF and exercise tolerance and higher NT-proBNP and NYHA class. Since previously evaluated biomarkers of endothelial health have failed to perform in this manner [10–12], the novel biomarker endocan has proven itself as a more reliable marker of endothelial status and HF severity in a chronic HF population.

At present, a considerable number of chronic HF patients continue to have a very poor quality of life and an unacceptable high risk of 1-year mortality, indicating that currently available treatment regimens remain inadequate in this fragile patient population [1–3]. In the current clinical setting, biomarkers play an important role in prognostic appraisal of HF and may also provide guidance on therapeutic approach [1, 4, 9, 10, 12, 23–25]. Our results suggest that endocan levels are associated with adverse prognosis in chronic HF, with every increase in endocan levels for 1 ng/mL being associated with a ~1,5-fold increase in HF-related events in patients deemed clinically stable and optimally managed. Importantly, in multivariate analysis, endocan emerged as an independent predictor of HF-related adverse events even after allowing for age, gender, ischemic vs. nonischemic etiology, LVEF, exercise tolerance, NT-proBNP, and NYHA class. In this manner, our work clearly shows that endocan is an independent predictor of HF-related mortality and hospitalization regardless of the underlying etiology, LVEF, exercise tolerance, NYHA class, and NT-proBNP levels and thus related to endothelial health, deteriorated by chronic HF itself. These data suggest that endocan could provide a prognostic benefit in individuals suffering from chronic HF and may potentially—in combination with already well-established clinical prognostic parameters—help to reliably identify the best target group within the chronic HF patient population which would mostly benefit from intensified medical treatment, stringent follow-up, and/or advanced heart failure management options.

Although our study has identified increased levels of endocan as independent predictors of chronic HF prognosis, some limitations to our work should be addressed. Firstly, only optimally managed chronic HF patients were included in the study; thus, our results cannot be generalized to all individuals suffering from various other stages of HF. Secondly, in our multivariate model, endocan was assessed only against most known and clinically established predictors of HF prognosis and not against other possible cofounders due to a relative small number of included patients. This is particularly important in terms of exercise capacity which was assessed with the 6MWT. Although 6MWT has been validated as an important prognostic factor in patients with HF [28], a comprehensive cardiopulmonary exercise test—providing maximal oxygen consumption as well as several other prognostic ventilator parameters—is today considered the method of choice for prognostic assessment and pretransplant risk assessment in the HF population [29, 30]. Thus, appraising the prognostic impact of endocan against 6MWT represents a major limitation of our study. Thirdly, our study does not provide the exact mechanism of the complex interplay between HF and endocan; thus, further research addressing this issue is anticipated.

## 5. Conclusion

In conclusion, our study is the first to address the expression of endocan in the ambulatory chronic HF population. We have shown that endocan is markedly elevated and associated with disease severity as well as long-term prognosis in patients with HF. Endocan emerged as an independent prognostic marker of HF-related mortality and hospitalization requiring inotropic support in chronic HF individuals, even after allowing for clinically established predictors of HF-related events. Our results suggest that endocan may serve as a simple marker for better risk stratification in the chronic HF population.

## Figures and Tables

**Table 1:** Baseline clinical and laboratorial characteristics of patients included in the study.

	All patients *n* = 120	Event *n* = 50	Event-free *n* = 70	*p*
Age (years) (mean ± SD)	71 ± 11	72 ± 11	70 ± 11	0.745
Gender (male), *n* (%)	77 (64)	37 (74)	43 (61)	0.172
HF etiology, *n* (%)				
Ischemic	61 (51)	31 (62)	31 (44)	0.018
Nonischemic	59 (49)	19 (38)	39 (56)	
LVEF (%) (mean ± SD)	36 ± 12	32 ± 9	38 ± 13	<0.001
NYHA class, *n* (%)				
II	77 (64)	23 (46)	53 (76)	0.001
III	43 (36)	27 (54)	17 (24)	
6 MWT (m) (mean ± SD)	262 ± 152	205 ± 140	292 ± 150	0.002
MLHF (pt) (mean ± SD)	37 ± 22	40 ± 23	35 ± 21	0.203
KI (%)	52 (43)	25 (50)	27 (39)	0.182
DM (%)	48 (40)	23 (46)	25 (36)	0.128
AH (%)	84 (70)	36 (72)	48 (69)	0.746
PAD (%)	22 (18)	8 (16)	14 (20)	0.517
HLP (%)	70 (58)	27 (54)	43 (61)	0.479
Therapy, *n* (%)				
RAAS inhibitors	120 (100)	50 (100)	70 (100)	N/A
MRA	83 (69)	36 (72)	44 (63)	0.573
*β*-Blockers	109 (91)	43 (86)	66 (94)	0.124
Diuretics	80 (67)	45 (90)	38 (54)	0.004
Antithrombotic	83 (69)	38 (76)	47 (67)	0.377
Statins	46 (38)	18 (36)	27 (38)	0.757
NT-proBNP (pg/mL) (median (IQR))	1967 (731-4352)	3595 (1817-7436)	1539 (602-3308)	<0.001
Endocan (ng/mL) (median (IQR))	3.38 (2.46-4.81)	4.26 (3.16-6.13)	3.21 (2.25-4.45)	<0.001

HF: heart failure; LVEF: left ventricle ejection fraction; NYHA class: New York Heart Association class; 6MWT: 6-minute walking test; MLHF: Minnesota Living with Heart Failure Questionnaire; KI: kidney insufficiency; DM: diabetes mellitus; AH: arterial hypertension; HLP: hyperlipidemia; RAAS inhibitors: renin-angiotensin-aldosterone system inhibitors; MRA: mineralocorticoid-receptor antagonists; *β*-blockers: beta blockers; NT-proBNP: N-terminal pro-b-type natriuretic peptide.

**Table 2:** Endocan levels in chronic HF patients in regard to patient's comorbidities.

Comorbidity		*N* (%)	Endocan levels (median (IQR))	*p*
CAD	Yes	57 (47)	3.37 (2.39-4.76)	0.976
	No	63 (53)	3.38 (2.48-4.85)	
PAD	Yes	22 (18)	3.61 (2.32-4.97)	0.218
	No	98 (82)	3.38 (2.48-4.85)	
DM	Yes	48 (40)	3.51 (2.55-4.50)	0.776
	No	72 (60)	3.25 (2.40-4.89)	
AH	Yes	84 (70)	3.30 (2.34-4.75)	0.292
	No	36 (30)	3.66 (2.82-5.01)	
HLP	Yes	70 (58)	3.25 (2.28-4.28)	0.401
	No	50 (42)	3.92 (2.58-5.31)	
KI	Yes	52 (43)	3.55 (2.44-4.97)	0.309
	No	68 (57)	3.26 (2.39-4.47)	

CAD: coronary artery disease; PAD: peripheral artery disease; DM: diabetes mellitus; AH: arterial hypertension; HLP: hyperlipidemia; KI: kidney insufficiency.

**Table 3:** Univariate and multivariate predictors of HF-related mortality and hospitalization requiring inotropic support.

	Univariate HR (95% CI)	*p*	Multivariate HR (95% CI)	*p*
Age (per 1 year increase)	0.979 (0.842-1.012)	0.289	0.993 (0.949-1.039)	0.761
Gender (male vs. female)	1.462 (0.710-3.012)	0.303	1.184 (0.478-2.936)	0.715
Etiology (ischemic vs. nonischemic)	1.615 (1.094-2.387)	0.016	1.445 (0.955-2.189)	0.082
LVEF(per 1% increase)	1.032 (1.001-1.062)	0.040	1.011 (0.978-1.045)	0.506
NYHA class	3.419 (1.877-6.231)	0.001	2.825 (1.299-6.144)	0.009
Log NT-proBNP	5.016 (2.587-9.725)	<0.001	2.207 (1.002-4.861)	0.049
6 MW (per 1 meter increase)	0.997 (0.995-0.999)	0.001	0.997 (0.995-1.000)	0.032
Endocan levels (per 1 ng/mL increase)	1.518 (1.269-1.816)	<0.001	1.471 (1.183-1.829)	0.001

HR: hazard ratio; CI: confidence interval; LVEF: left ventricle ejection fraction; NYHA class: New York Heart Association class; 6MWT: 6-minute walking test; log NT-proBNP: logarithmic value of N-terminal pro-b-type natriuretic peptide.

## Data Availability

The data used to support the findings of this study are available from the corresponding author upon request.
